# Glimpsing the Impact of COVID19 Lock-Down on People With Epilepsy: A Text Mining Approach

**DOI:** 10.3389/fneur.2020.00870

**Published:** 2020-08-19

**Authors:** Jacopo Lanzone, Cristina Cenci, Mario Tombini, Lorenzo Ricci, Tommaso Tufo, Marta Piccioli, Alfonso Marrelli, Oriano Mecarelli, Giovanni Assenza

**Affiliations:** ^1^Unit of Neurology, Neurophysiology, Neurobiology, Department of Medicine, University Campus Bio-Medico of Rome, Rome, Italy; ^2^DNM-Digital Narrative Medicine, Rome, Italy; ^3^Policlinico Gemelli Foundation University Hospital IRCSS, Rome, Italy; ^4^Unit of Neurology, AO S. Filippo Neri, Rome, Italy; ^5^Unit of Neurology, AO S. Salvatore, L'Aquila, Italy; ^6^Department of Neurology and Psychiatry, “Sapienza” University of Rome, Rome, Italy

**Keywords:** COVID-19, epilepsy, text-mining, neuropsychology, natural-language processing

## Abstract

**Objectives:** To describe how the recent lock-down, related to SARS-COV-II outbreak in Italy, affected People With Epilepsy (PwE), we designed a survey focused on subjective reactions. Using Natural Language Processing (NLP), we analyzed words PwE and People without Epilepsy (PwoE) chose to express their reactions.

**Methods:** As a subset of a larger survey, we collected from both PwE (427) and PwoE (452) single words (one per subject) associated to the period of lock down. The survey was spread thanks to the efforts of Italian league against epilepsy Foundation during the days of maximum raise of the pandemic. Data were analyzed via bag of word and sentiment analysis techniques in R.

**Results:** PwoE and PwE showed significantly different distribution in word choice (X^2^, *p* = 4.904e−13). A subset of subject used positive words to describe this period, subjects with positive feelings about the lock down were more represented in the PwE group (X^2^, *p* = 0.045).

**Conclusion:** PwoE developed reactive stress response to the restrictions enacted during lock-down. PwE, instead, chose words expressing sadness and concern with their disease. PwE appear to internalize more the trauma of lock down. Interestingly PwE also expressed positive feelings about this period of isolation more frequently than PwoE. Our study gives interesting insights on how People with Epilepsy react to traumatic events, using methods that evidence features that do not emerge with psychometric scales.

## Introduction

In order to describe the impact of epileptic disorders on People With Epilepsy (PwE) we often take advantage of quantitative scores such as psychometric scales targeting depressive symptoms, emotion dysregulation, anxiety and stigma perception (1). Scores do not take into consideration qualitative and more subjective facets of epilepsy. In this brief communication we report how we used Natural Language Processing (NLP) to better describe differences between People With-out Epilepsy (PwoE) and PwE in coping with the recent SARS-CoV-2 pandemic.

NLP methods are widely used in marketing and social sciences but they are under-represented in the study of chronic medical conditions such epilepsy (2–4). We think that language processing can be useful in describing interesting aspects of coping with chronic diseases such as epilepsy (4, 5).

## Methods

We collected word clusters as a subset of a broader online survey on COVID-19 and epilepsy (6, 7). The survey was spread thanks to the efforts of LICE [Lega Italiana Contro l'Epilessia, the Italian chapter of the International League Against Epilepsy (ILAE)] Foundation and included clinical data and psychometric scales.

Respondents were asked to type a single simple word they came across when thinking about how the lock-down caused by COVID19 pandemic affected their life.

Data, consisting in a single word for each response, was imported in R as a commas separated vector (csv) file and processed with text mining libraries (Tidytext), using a “bag of words” approach (8).

Answers were stemmed, transformed to upper-case and collected in a digital corpus that was then subset among PwE and PwoE groups. Single words were translated in English using Google cloud^TM^ translations and were manually controlled by the authors. Translation was considered to be robust since terms used were simple and generally non-metaphorical. Singletons (single occurring words) were eliminated, since they do not bear interesting information. To evidence differences in occurrence of the most used terms we considered terms with at least three recurrences in one of the groups (PwoE and PwE) and created a difference matrix of words occurrence in the two groups.

Differences were thus calculated on a reduced dataset (excluding single occurring word and words with frequency <3). Moreover, we calculated polarization score using the “Affin” lexicon (9). Polarization is a technique used in sentiment analysis that leverages lexicons: large libraries of words assigned with positive or negative value depending on the polarity of the term.

Differences in word frequency distribution and difference in distribution of positive and negative words were tested with Chi^2^ Difference in polarity were tested with Mann-Whitney test.

Alpha level was set as *p* = 0.05 for statistical significance.

## Results

Our survey opened on April 11, 2020 and closed at 11.59 p.m. of April 16, 2020. The survey was completed by 879 subjects:427 PwE (327 females, 38.6 ± 11.8 years) and 452 PwoE (331 females, 43.89 ± 12.25 years). Difference in age and sex between the two groups was not significant, women were more represented than men [as is described that they tend to be more keen to answering online surveys (10)]. Data on psychometric scales and in-depth clinical data was published elsewhere (7). Among PwE 49.6% (212/427) were seizure free and 15.7% (67/427) reported seizure worsening during the lockdown period (7), these categories were too unbalanced and we did not find significant difference in word choice distribution among them.

After eliminating singletons and words with <3 occurrences our corpus consisted of 605 entries: 46.6% PwE (282/605), 53.4% PwoE (323/605).

Chi^2^ test showed significant difference in the frequency distribution between the word used by the two groups (X^2^ = 159.06, df = 51, *p* = 4.904e−13).

Chi^2^ test showed increased frequency in the occurrence of words with positive “Affin” score in the PwE group compared to the PwoE (X^2^ = 5.3953, df = 1, *p* = 0.045, PwE = 10.2%, 29/282; PwoE = 5.2%, 17/323).

We analyzed polarization scores among the two datasets, finding no significant differences and an average polarization value of −1.28 in PwE and −1.39 in PwoE (polarization range −5, 5).

**Figure 1:**
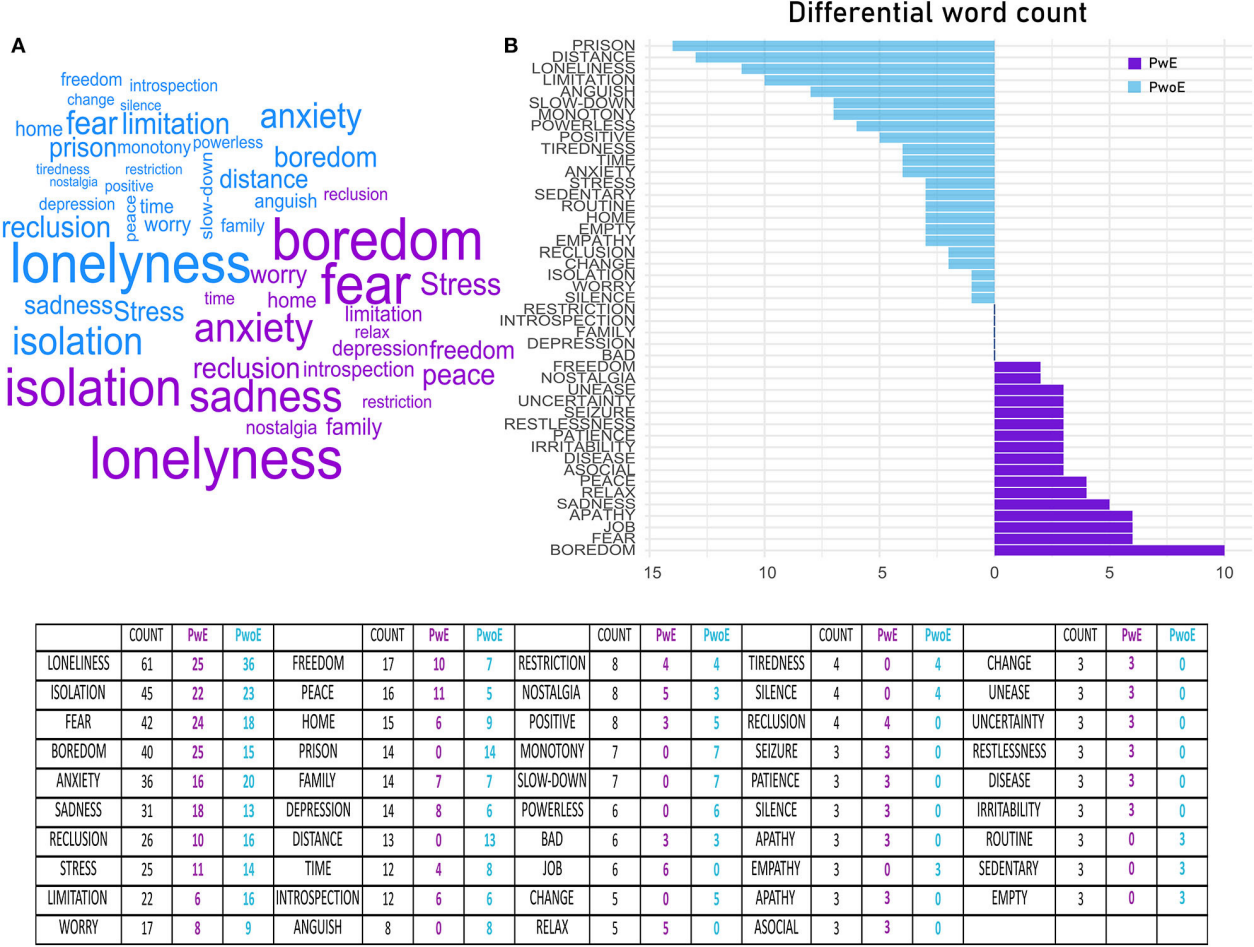
Depicts a word-cloud based on word counts in the two groups PwE (purple) and PwoE (blue) **(A)**. Segment **(B)** shows the differential frequency of word counts between the two groups, thus evidencing terms that are over-expressed in PwoE (blue) or in PwE (purple). The table shows absolute and group word frequency. PwoE in blue (People without Epilepsy), PwE in purple (People with Epilepsy).

Using the word corpus, we created (Figure 1A) that is a “mind-map” of feelings and emotions related to the lock down in both groups. In Figure 1B, we show the difference in word frequency between the two groups, highlighting terms that are over expressed and under expressed in the PwoE and PwE groups.

## Discussion

Textual analysis helps to evidence interesting patterns in word choice. PwE and PwoE tend to use different words to describe the lockdown period. While the most expressed words are the same (Figure 1A), words that are over expressed in the PwE, and in PwoE point out how the two groups cope differently with the same stressful event.

PwoE consistently over-report many terms that express anxiety as a reactive response to the stressful event; words like “prison,” “distance,” “loneliness,” “anguish,” “stress,” “change” are frequently used. In our interpretation, PwoE develop anxiety since they are concerned with practical issues and limitations of lock down.

On the other hand, PwE tend to over-report terms like “fear” “boredom” “sadness” “apathy” “asocial” “disease” “seizure”; these terms are related to something more than reactive stress. PwE during lockdown do not just feel isolated, limited and anxious in their day-to-day life; they also worry about their disease and tend to develop depressive thoughts.

This could be partly related to the well-known fact that PwE tend to be more depressed than PwoE (11–13), but could also relate to the heavy burden of stigma in PwE.

Our hypothesis is that while PwoE tend to react to isolation as expected with anxiety, PwE already feel as they live in a condition of relative stigma and isolation (14) and thus tend to give a more negative interpretation to the lock down, developing feelings in the depression sphere (15, 16).

Moreover, it is interesting to note that both in PwoE and PwE there is a subgroup of people expressing positive feelings about the lock down. This occurs more frequently in PwE as is shown by less negative scores in average polarization of the terms used and significant Chi2.

Apparently, some see the lockdown as a chance to “relax” and find “peace.” In our interpretation PwE express more this feeling since isolation reliefs many of them from the social burden of their disease. This condition of forced isolation (lock-down) can be interpreted by some as a form of leveling of the stigma and pressure usually perceived by epileptic people in their ordinary life. Therefore, PwE report more frequently relief during this moment of temporary interruption.

## Conclusion

We report results from an exploratory text mining study on how PwE and PwoE cope with the lock-down related to SARS-CoV-2. PwoE respond to the lock-down developing reactive anxiety while PwE seem to internalize this stressful event, developing feelings that lay in the depressive sphere. Moreover, some individuals reported relief in this period of isolation, these subjects are more represented in the PwE group.

## Limitations

Due to privacy regulation we could not control the exact provenience of each answer, this could be a source of selection bias. Due to the nature of NLP our study is more descriptive than inferential thus is more helpful in making hypothesis from large set of data.

## Data Availability Statement

The raw data supporting the conclusions of this article will be made available by the authors, without undue reservation.

## Ethics Statement

Ethical review and approval was not required for the study on human participants in accordance with the local legislation and institutional requirements. The patients/participants provided their written informed consent to participate in this study.

## Author Contributions

JL analyzed data and wrote the full manuscript. GA, OM, and CC helped in developing the surveys and interpreting the results and supervised the analysis process. MT, LR, TT, MP, and AM helped developing the surveys and greatly helped in spreading the survey among patients and controls. All authors contributed to the article and approved the submitted version.

## Conflict of Interest

The authors declare that the research was conducted in the absence of any commercial or financial relationships that could be construed as a potential conflict of interest.
